# Educating Residents in Abdominal Wall Closure: An Overview

**DOI:** 10.3389/jaws.2023.12159

**Published:** 2023-12-01

**Authors:** Justin Leavitt, Matthew Hager, Colston Edgerton, W. Borden Hooks, William Hope

**Affiliations:** Department of Surgery, Novant Health New Hanover Regional Medical Center, Wilmington, NC, United States

**Keywords:** education, hernia, prevention, laparotomy, closure, simulation, resident

## Abstract

**Background and Aims:** Incisional hernia prevention has become an important concept for surgeons operating on the abdominal wall. Several techniques have been proposed to help decrease incisional hernia formation with suture closure of the abdominal wall being one of the cornerstones. Technical details that have been reported to decrease incisional hernia rates include achieving a 4:1 Suture to Wound length ratio and the use of a small bites technique. Despite evidence to support many of these techniques there appears to be a gap in practice patterns amongst practicing surgeons. Introducing and promoting these principles in surgical residency may help to close this gap. This paper reviews our experience with surgical training for abdominal wall closures at our institution.

**Materials and Methods:** Programs and projects related to abdominal wall closure were reviewed from our institution from 2010-Present. Type of project, intervention, and impact on education was evaluated and summarized.

**Results:** Seven projects were identified relating to surgical training and abdominal wall closure. Three projects dealt with skills training using an abdominal wall simulation model and related to suturing techniques. Two projects were clinical studies focused on suture to wound length ratios and improving outcomes with this variable in a residency training program. Two projects dealt with models relating to abdominal wall closure and education.

**Conclusion:** Implementation of educational programs in surgical residency programs can lead to improvements in technique and knowledge around abdominal wall closure and help in research endeavors.

## Introduction

**FIGURE 1:**
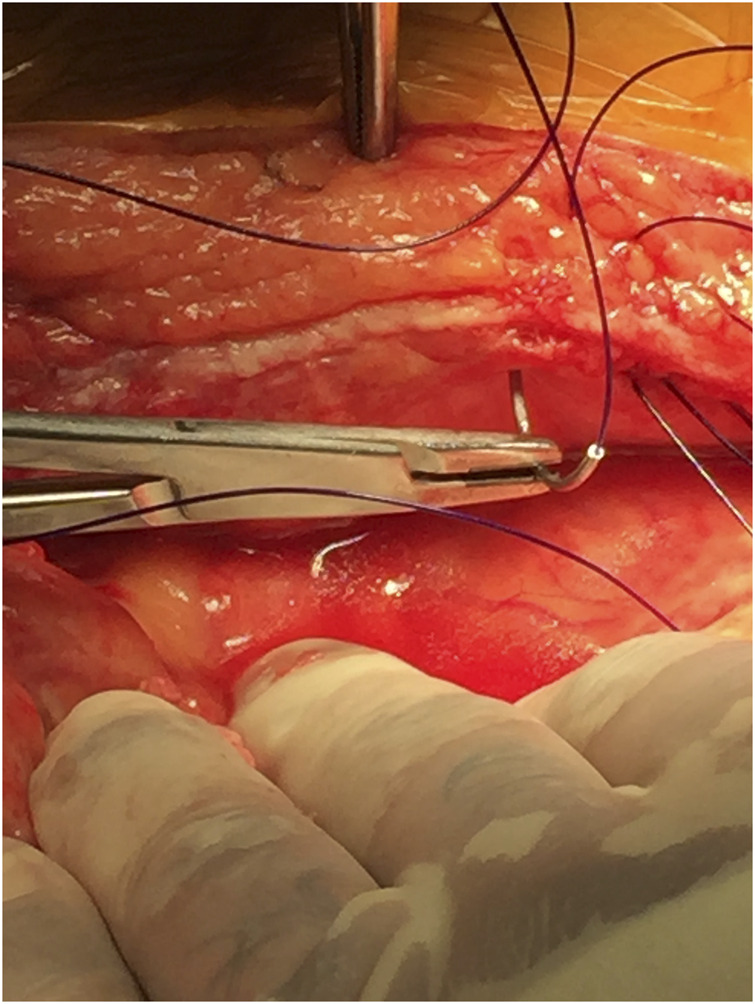
Small-bite technique of fascial closure using running polydioxanone (PDS) suture.

The incidence of ventral hernia following laparotomy has been shown to be 12.8% within 2 years of surgery [1], often leading to further re-operation [2], complications, and decreased quality of life. These outcomes have led hernia prevention with an emphasis placed on surgical technique to be an important topic to mitigate morbidity from ventral hernias. Although the formation of incisional hernias after laparotomy are multifactorial in origin, the approach to laparotomy closure is one area where surgeons can have a major impact. We have seen recommended techniques change over the last decade and eventually become implemented in society guidelines [3]. Two prominent changes were the 4:1 suture-to-wound length (S:W), and the small bite (5 mm) techniques (Figure 1).

The utilization of these recommendations varies. Respondents to a survey of American Hernia Society (AHS), European Hernia Society (EHS), and International Hernia Collaboration (IHC) members indicated that 63% performed 4:1 S:W technique, and 58% used small-bite techniques [4]. The reasons for not using these techniques include not being familiar with the techniques, not being applicable to practices, or the recommended techniques take too long. In a prospective study, surgeons and trainees performed small-bite techniques in only 30.7% of cases per suture length analysis following an online course, despite a majority responding to a 1 year follow-up survey supporting the technique [5].

To ensure the best current practices of abdominal wall closure, we have undertaken several approaches to assess and teach these contemporary techniques to residents at our institution. This paper reviews some of these methods and their potential improvements.

## Materials and Methods

We reviewed all programs and projects related to abdominal wall closure in our surgical residency program from 2010-Present. Programs could be didacts or skill related programs but had to encompass education related to laparotomy closure. Type of project, intervention, and impact on education was evaluated and summarized.

## Results

**FIGURE 2:**
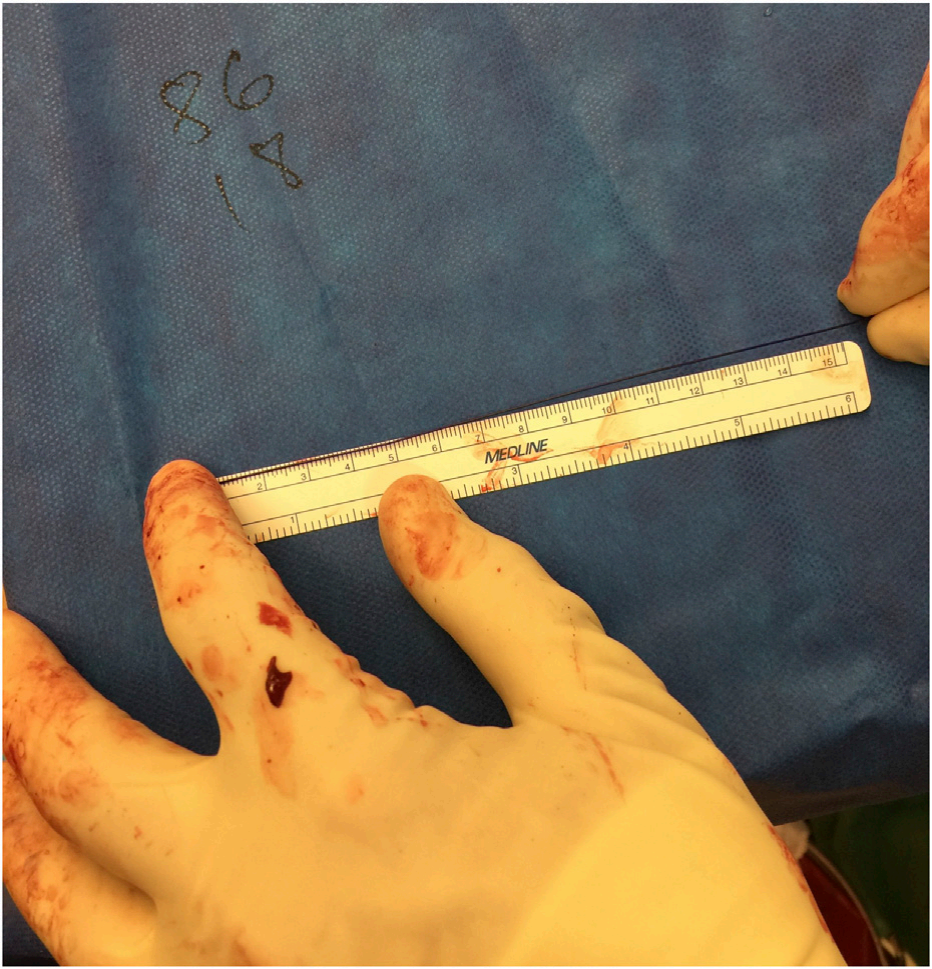
Measuring suture remainder after fascial closure to calculate suture to wound length ratio.

Seven projects were identified related to surgical training and abdominal wall closure. Several utilized simulation models, however, two of the studies focused on the models themselves. The first described the types, strengths, and weaknesses of various available models. In the second study, we constructed a model for complex hernia repair simulation. Three projects were audits of resident knowledge of fascial closure techniques and current guidelines, followed by technical demonstration on a simple fascial layer model. The final two projects described adherence to a 4:1 suture to wound length ratio during actual cases. The first of these two projects followed an attending surgeon for 100 consecutive cases where suture and wound lengths were measured and recorded (Figure 2). The second project was similar, with a pre- and post-intervention of resident training to evaluate adherence to the recommended suture to wound length ratio.

**FIGURE 3:**
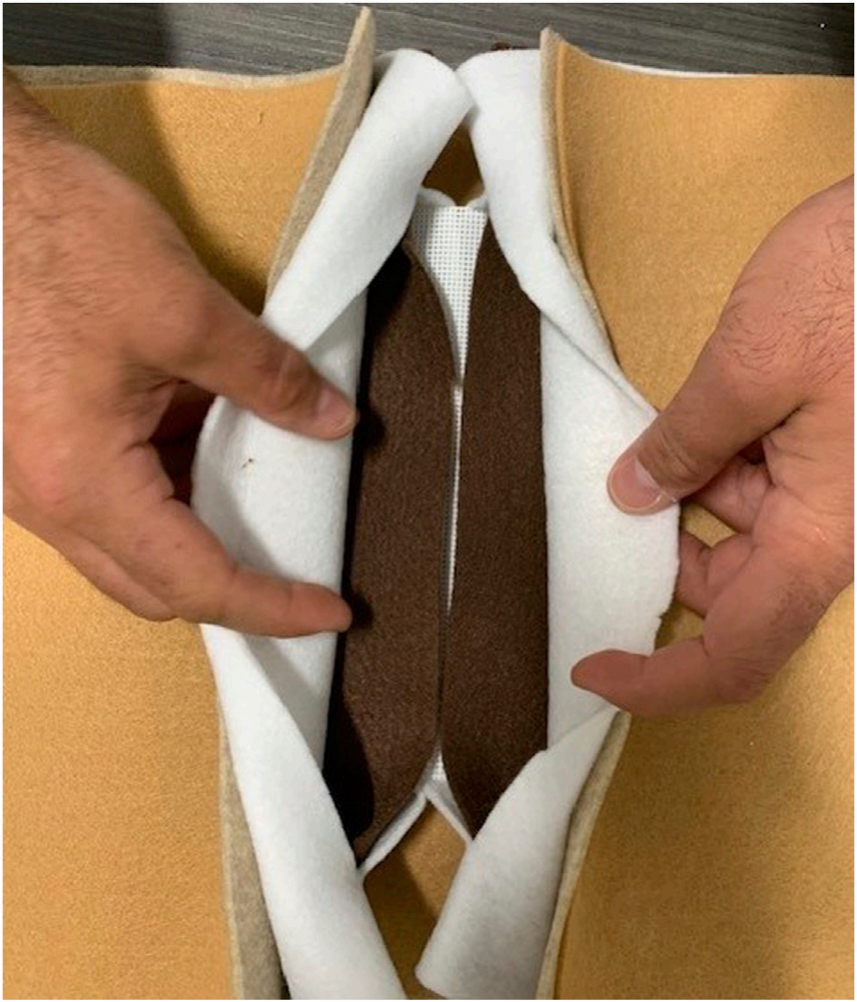
Abdominal wall model with layers of the wall represented by felt with different thickness and characteristics, providing an affordable model for fascial closures and plane development.

An important tool for teaching surgical residents fascial closure techniques is a good simulation model of the abdominal wall. Our group reviewed available training modules and reported on the strengths and weaknesses of the available models which include human and animal, synthetic, and virtual reality [6]. Naturally, human cadaver and porcine models provide the most realistic simulation, however, are not feasible for routine or impromptu training. We have developed a scalable approach with a very inexpensive and simple felt model for simulating laparotomy closure which works well for focused training on single layers of fascial closure [7]. We have also developed a more sophisticated model that more accurately approximates the layered anatomy of the abdominal wall which allows for more advanced training on plane development, component release, and mesh placement [8] (Figure 3).

Three of our projects have assessed resident familiarity and technique with implementing the recommended suture length and bite-length techniques. The first project was an audit of surgical resident knowledge of laparotomy closure and fascial closure skill session. This study reported that improvement in skill increased with experience, but the technical aspect of closure was obtainable at all training levels. The report found that many residents had little knowledge of suture to wound length ratio and other principles of laparotomy closure. These findings guided future education and studies [9].

The second used similar methods and modeling with a new cohort of surgical residents and were compared to their OB/GYN colleagues due to reported differences of training curriculums. We found a significant difference in familiarity of recommendations for suture-to-wound length and bite sizes based off training program [10].

**FIGURE 4:**
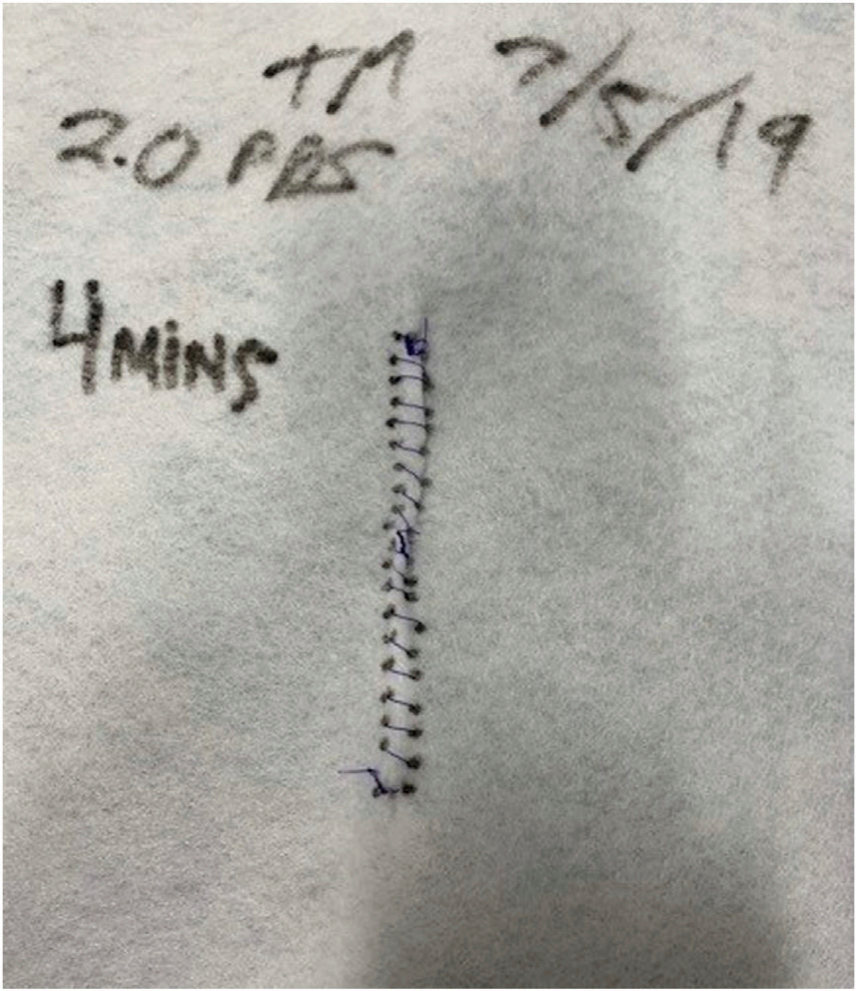
This piece of felt was secured to a wooden frame with a “midline” incision to evaluate the time it took residents to run a simulated fascial closure using 5 mm bites with 5 mm travel. The bite-size and travel distance were measured and analyzed for accuracy.

The last simulation study evaluated the ability to perform the small bite fascial closure technique. This found that residents performed a more uniform and consistent closure when asked to use the small bites technique compared to the 1 cm bite and 1 cm advance technique. This study also showed an improvement in accuracy when templates were used to guide technique compared to standard suturing without a template [7] (Figure 4).

**FIGURE 5:**
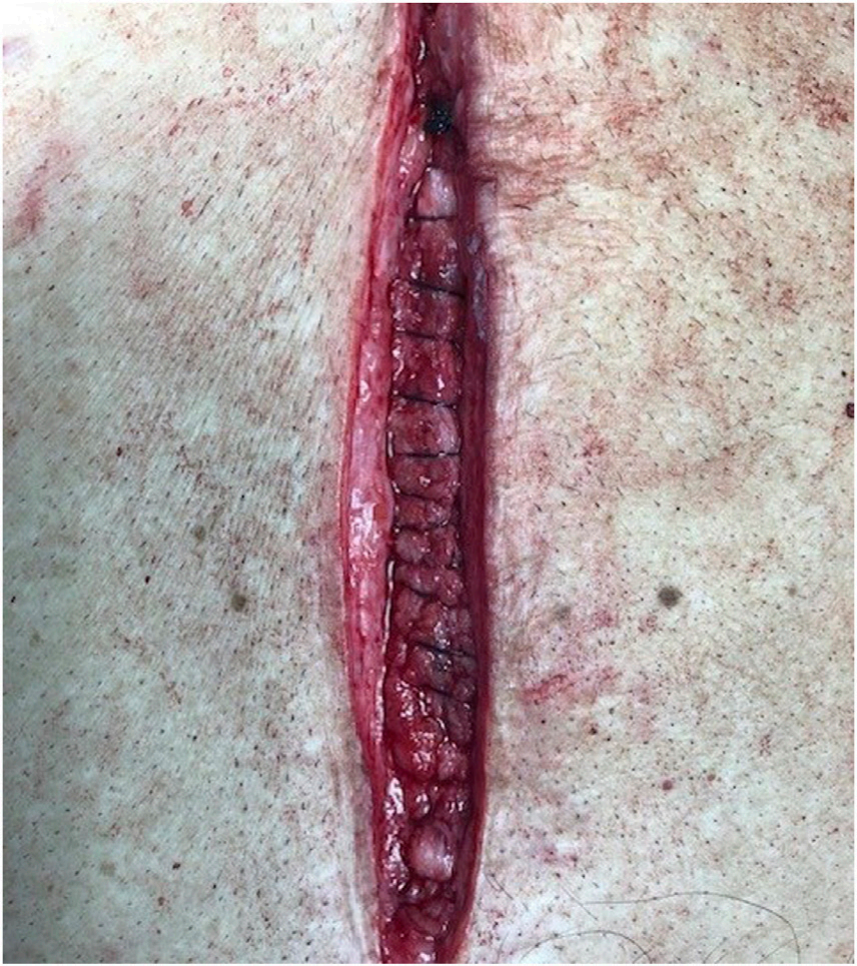
Fascial closure with approximately 5 mm bites and 5 mm travel.

Two projects were clinical specifically related to laparotomy closure and suture to wound length ratios. The first project was a prospective study of 100 consecutive abdominal wall closures examining how well the surgeon adhered to a 4:1 suture to wound length ratio (Figure 5). This study reported a 76% adherence rate to achieving a 4:1 suture to wound length ratio with the only reported risk factor for not achieving was when two residents closed the abdomen compared to one resident/attending closure [11].

A follow up study was performed examining the subsequent 100 abdominal wall closures after the intervention of evaluating the accuracy of closure with 4:1 ratio. This post-intervention assessment included 200 consecutive abdominal wall closures and showed an improvement in the achievement of a 4:1 suture to wound length ratio from 76% (before) to 92% (after). It was also reported that having 2 residents involved in closure no longer represented a risk factor for not achieving a 4:1 ratio after the education intervention, demonstrating the role in technical education to improve closure techniques [12].

## Discussion

Our review highlights the role of education and simulation on surgical resident training to a specific topic of hernia prevention. By focusing on an important technical topic that has clear impact on outcomes, which can be easily measured and universally applied across specialties, we have been able to audit, educate, and evaluate our knowledge in this area over the past 10 years.

We found that an audit of trainee knowledge is likely the best place to start and will likely differ for trainees from different specialties and levels. After gaining this basic knowledge, the next step for programs wanting to implement a training program is to choose a simulation model that meets their needs. As mentioned in our review, there are several models to choose from for abdominal wall closure ranging from simple, low-cost models to more expansive animal and virtual reality models. For our purposes, we focused on models that were affordable, reproducible, simple, and accessible. The realism of the model is balanced with the expense and difficulty to create and maintain. The audits of resident ability utilized a very simple single layer of material that was sufficient to evaluate bite width, travel, and knot tying. The second model described had multiple components and intended for complex hernia repair simulation, but still cost around $10 and took a single day to assemble using a combination of a plastic sheet, felt, and other fabrics available from our local craft store. Both models are easy to repair, replace, and modify.

A model teaching laparoscopic port insertion achieved a similar balance of cost and fidelity using a combination of soft foam, vinyl and floor pads to reconstruct the abdominal wall [13]. Another demonstrated that med students preferred suture practice on a dry sponge similar to our felt model, rather than using pigskin, oranges and other materials [14]. Despite these examples, in comparison to laparoscopic and robotic surgery, our examination coincides with a prior review finding a paucity of literature describing open surgical simulation [15]. We suspect this may partially be due to a common “do-it-yourself” approach to open simulation projects, and a lack of standardized curriculums such as the Fundamentals of Laparoscopic Surgery program.

While there are several standardized curriculums for fascial closure, they have not focused on current surgical principles such as suture to wound length ratio and the short stitch techniques. These principles, however, are easy to learn and teach with instructional videos available and multiple surgical articles that could be used for journal clubs. The ultimate goals are to combine a didactic and simulation module that will help translate skill for fascial closure into the operating room.

There are many limitations in our studies. Our residency is a small community program and thus we have small sample sizes, though our training is likely similar to other larger and academic programs using the standard “rectangular” residency model. Hopefully, with increased emphasis, more programs will engage to fascial closure education and potentially lead to a national or international curriculum that could be used by all trainees. While the development of a training curriculum at our program has improved adherence to current principles of bite travel and size as demonstrated in the studies evaluating real world performance, the focus of these studies is on technique outcomes and do not measure how this translates to other surgical outcomes. Our simulated abdominal wall model has many advantages but does not compare to the complexity of a real abdominal wall with some of the challenges associated.

In conclusion, we have conducted several educational studies performed in our surgery residency program relating to fascial closure. These have demonstrated that it is possible to improve awareness and knowledge of important principles of fascial closure and have described a low-cost abdominal wall model that can be used for simulation. We have also reported improved clinical adherence to the principles of obtaining a 4:1 suture to wound length ratio with improved education. Lastly, we have explored some potential advancements for fascial closure such as the idea of templating closure to allow for improved accuracy.
